# Fat of the Gut: Epithelial Phospholipids in Inflammatory Bowel Diseases

**DOI:** 10.3390/ijms222111682

**Published:** 2021-10-28

**Authors:** Lidiya V. Boldyreva, Maryana V. Morozova, Snezhanna S. Saydakova, Elena N. Kozhevnikova

**Affiliations:** 1 Scientific-Research Institute of Neurosciences and Medicine, 630117 Novosibirsk, Russia; asd@mcb.nsc.ru (L.V.B.); morozova.maryana@mail.ru (M.V.M.); custodian.of.midnight@gmail.com (S.S.S.); 2 Institute of Molecular and Cellular Biology, The Siberian Branch of the Russian Academy of Sciences, 630090 Novosibirsk, Russia; 3 Siberian Federal Scientific Centre of Agro-BioTechnologies of the Russian Academy of Sciences, 630501 Krasnoobsk, Russia; 4 Novosibirsk State Agrarian University, 630039 Novosibirsk, Russia

**Keywords:** IBD, Crohn’s disease, ulcerative colitis, phosphatidylcholine, lysophosphatidylcholine, phosphatidylethanolamine, phosphatidylserine, phosphatidylinositol, phosphatidic acid, lysophosphatidic acid

## Abstract

Inflammatory bowel diseases (IBD) comprise a distinct set of clinical symptoms resulting from chronic inflammation within the gastrointestinal (GI) tract. Despite the significant progress in understanding the etiology and development of treatment strategies, IBD remain incurable for thousands of patients. Metabolic deregulation is indicative of IBD, including substantial shifts in lipid metabolism. Recent data showed that changes in some phospholipids are very common in IBD patients. For instance, phosphatidylcholine (PC)/phosphatidylethanolamine (PE) and lysophosphatidylcholine (LPC)/PC ratios are associated with the severity of the inflammatory process. Composition of phospholipids also changes upon IBD towards an increase in arachidonic acid and a decrease in linoleic and a-linolenic acid levels. Moreover, an increase in certain phospholipid metabolites, such as lysophosphatidylcholine, sphingosine-1-phosphate and ceramide, can result in enhanced intestinal inflammation, malignancy, apoptosis or necroptosis. Because some phospholipids are associated with pathogenesis of IBD, they may provide a basis for new strategies to treat IBD. Current attempts are aimed at controlling phospholipid and fatty acid levels through the diet or via pharmacological manipulation of lipid metabolism.

## 1. Introduction

Inflammatory bowel diseases (IBD) are inflammatory pathologies of the gastrointestinal tract, with Crohn’s disease (CD) and ulcerative colitis (UC) being the two major types. CD affects the small intestine and large intestine, in addition to the mouth, esophagus, stomach and anus, and is characterized histologically by transmural inflammation, noncaseating granulomas and thickened submucosa. UC primarily affects the colon and the rectum, and is characterized by superficial damage to the mucosa, which is followed by cryptitis and crypt abscesses [1]. The prevalence rates of IBD have a different geographic distribution, with high rates in Europe and North America and low rates in Asia [2]. It is important that an increase in the incidence of IBD and a decrease in the onset age up to childhood are observed [3].

IBD persist to be chronic, relapsing disorders with complex etiology. The risk factors comprise genetic predisposition, environmental stress, diet and intestinal microbiota composition [4,5]. About 200 genetic loci were identified as IBD-linked, including major inflammatory genes that may promote an exaggerated immune response in the intestine [6,7,8,9]. However, the onset of the disease, its characteristics and severity can be modulated by additional risk factors such as a Western diet, smoking and/or alcohol abuse [10,11]. Thus, IBD development is a multifactor process with a complex therapy. To date, therapeutic strategies are mainly confined to bringing patients into stable remission, with no chance of a complete cure. Because IBD are accompanied by the synthesis and release of inflammatory cytokines, aminosalicylates and anti-inflammatory drugs, such as steroids, glucocorticoids and immune suppressors, remain the most effective IBD therapies [12,13]. Despite significant progress in understanding the molecular mechanisms behind IBD and improvement in therapeutic strategies, about 20–30% of patients with UC still need surgery [14,15]. Therefore, the search and implementation of new treatments for IBD remains highly relevant.

The intestinal epithelium has a surface area of approximately 400 m^2^ made of a single layer of intestinal epithelial cells (IECs) largely adapted to perform metabolic and digestive functions. IECs present the rapidly proliferating monolayer with a complete turnover of 24 to 96 h [16]. The proliferative compartment of the epithelium is localized at the bottom of an intestinal crypt followed by a gradient of increasingly differentiated epithelial cells along the vertical axis [17,18]. Healthy intestinal epithelium coordinates appropriate immune responses, including tolerance and pathogen specific immunity by innate and adaptive immune systems. Thus, IECs regulate both responses to luminal antigens and maintain intestinal homeostasis via a delicate balance between pro- and anti-inflammatory responses [19].

Under homeostatic conditions, a single layer of enterocytes provides an efficient physical, chemical and electrical barrier against luminal microbial community and external factors. Epithelial defense mechanisms can be categorized into three key components: pre-epithelial, epithelial and post-epithelial defense, the latter being represented by the lamina propria [20]. The pre-epithelial mucus barrier is composed of mucin associated with other proteins and lipids, and forms a continuous gel. A bicarbonate-rich fluid is secreted into the mucus, maintaining a neutralizing pH at the epithelial surface. When the integrity of the epithelium is compromised, luminal antigens, including pathobionts, invade the subepithelial compartment, thus activating and/or sustaining deregulated inflammatory immune responses [21,22]. An inflammatory trigger promotes the synthesis of inflammatory chemokines with a robust influx of neutrophils into the tissue within hours of damage [23]. Moreover, during the acute period of the inflammatory response, the mucosal milieu is enriched in inflammatory cytokines, metabolites of arachidonic acid and other pro-inflammatory mediators that activate the recruited leukocytes [24]. Once the inflammatory response has started, Tumor necrosis factor alpha (TNF-α), Interleukin one beta (IL-1β) and other inflammatory factors amplify the immune reaction leading to further damage of the intestinal mucosa [25,26].

One of the prominent features of IBD is the lack of balance between pro-inflammatory and anti-inflammatory responses, which results in an uncontrolled inflammation mediated by resident innate immune cells [27]. These cells promote inflammatory cytokines production by Th-cells, which in turn activate the Nuclear factor kappa B (NF-ƙB) signaling cascade in the epithelium, thus assisting defense mechanisms such as barrier alteration and adaptive immune responses. Under unfavorable conditions, intestinal bacteria or their cell wall components and toxins have unlimited access to the host immune system via a compromised intestinal barrier [28]. In addition to the commensal microflora, a number of pathogenic microorganisms may cause IBD [4]. This process further promotes inflammation, including adaptive immune response and autoimmunity, resulting in massive inflammation and tissue damage that no longer can be resolved via natural anti-inflammatory and wound-healing mechanisms. Long-term inflammation finally develops into a chronic form and results in tissue remodeling and substantial changes in cell physiology and metabolism.

As the primary function of the gastrointestinal tract is digestion and metabolism of nutrients, IECs pose multiple metabolic pathways that become deregulated upon inflammatory response. Alternatively, wide spectra of nutritional components act as regulators of cell signaling and metabolism, and substantially influence epithelial physiology [29]. One of the essential functions of the gastrointestinal tract is digestion and turnover of lipids, which constitute a major building material of cell membranes, an energy source, and hormone and signal transduction regulators [30]. Bile acids, cholesteryl esters, phospholipids and triacylglycerols are digested in the intestinal lumen, and taken up and re-synthetized in the enterocytes. Some of them are metabolized in the intestine and some are packed into chylomicrons and transferred into the lymph and blood. Short- and medium-chain fatty acids passively diffuse through the cell membrane [31]. Multiple studies report that lipid metabolism is impaired in UC and CD patients, which is further supported by animal models of IBD [32,33,34,35]. In recent years, it has become clear that inflammatory cytokines can regulate genes involved in glucose and lipid metabolism, affecting overall energy homeostasis [36]. In agreement with this, clinical and animal studies revealed that chronic inflammation is associated with reduced energy metabolism and strong mitochondrial damage, which results in reduced ATP production in the intestinal epithelium [37,38,39,40]. In turn, ATP depletion triggers intestinal barrier impairment and worsens the course of colitis [41]. The shape of mitochondria and number and structure of mitochondrial cristae are often altered in electron microphotographs of human and animal IBD models [42,43,44]. However, mitochondria are not the only membranous organelles that alter shape and physiology upon colitis. Our previous study, in addition to reports by other authors, revealed the abnormal shape of multiple membranous organelles, including mitochondria, endoplasmic reticulum (ER) and Golgi apparatuses, and cellular and nuclear membranes during inflammation [37,38,39,45,46,47]. These indicate that membrane-forming phospholipids may be substantially deregulated upon colitis, which is further supported by experimental data [48,49,50]. Indeed, clinical data suggest that some important phospholipids such as phosphatidylcholine may be downregulated in IBD, which seems to be the cause of ER damage and the inability to secrete mucin granules [51]. Knockout of cytidine triphosphate (CTP): phosphocholine cytidylyltransferase-α–a rate-limiting enzyme in the major pathway for PC biosynthesis results in colitis, lack of a mucus layer and intestinal barrier permeability. Ultrastructural changes in this mouse model strongly resemble those we observed upon direct knockout of the *Mucin-2* gene, including mitochondrial shape and microvilli damage [37,51]. Phosphatidylinositol (PI) derivatives are also implicated in the pathogenesis of IBD as signaling molecules with notable changes in the membranous organelle ultrastructure [52]. In addition to their role in membrane formation, phospholipids were shown to act separately within the nucleus as a part of chromatin and as signaling molecules with a potent role in regulation of gene expression [53]. Many phospholipids directly activate genes as ligands for nuclear receptors and regulate lipid and glucose homeostasis, steroid biosynthesis and cell proliferation [53,54,55]. Another important role of phospholipids is activating innate-like T-cells and regulation of the immune response, infection and cancer [56,57,58]. Alternatively, phospholipids may attenuate receptor function by altering membrane fluidity or by phospholipid composition within a membrane or lipid rafts [59,60]. Some phospholipids, such as phosphoinositides, and their modifying enzymes are asymmetrically distributed in the plasma membrane, and serve to discriminate apical functional domains in polarized epithelia [61,62]. Moreover, phospholipids control maintenance of stem cell identity via their uneven distribution on the plasma membrane, which assists asymmetric cell division [63]. Although many studies suggest versatile roles of phospholipids in multiple cellular processes, the involvement of phospholipids in IBD pathogenesis in terms of their bioactivity and therapeutic potential remain largely overlooked. Given that the role of metabolism in the regulation of cell fate and function has emerged in the past years, it seems appropriate to comprehend our current understanding of individual phospholipid turnover during IBD and their potential role as bioactive molecules.

## 2. Major IECs’ Phospholipids, Their Metabolism, the Role in IBD and Therapeutic Potential

**Figure 1:**
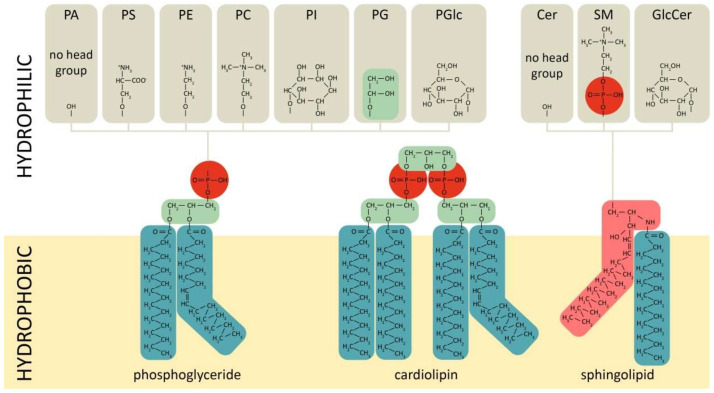
Chemical structures of main phospholipid types. Red—phosphate group, green—glycerol, pink—sphingosine, dark—green-fatty acids. PC/E/S/I/G/Glc—phosphatidylcholine/-ethanolamine/-serine/-inositol/-glycerol/-glucoside, PA—phosphatidic acid, SM—sphingomyelin, Cer—ceramide.

**Figure 2:**
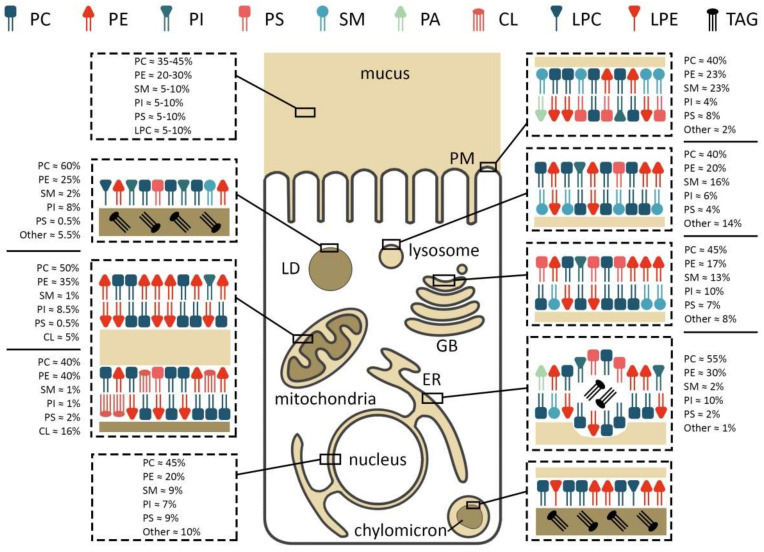
Membrane composition and localization of major phospholipids in an enterocyte. PC/PE/PS/PI—phosphatidylcholine/-ethanolamine/-serine/-inositol, LPC/E—lysophosphatidylcholine/-ethanolamine, TAG—triacylglycerol, SM—sphingomyelin, CL—cardiolipin, PM—plasma membrane, LD—lipid droplet, GB—Golgi body, ER—endoplasmic reticulum.

Phospholipids comprise a class of complex lipids containing a phosphate group and two fatty acid derivatives linked by a glycerol molecule (Figure 1). The phosphate group represents a hydrophilic head, whereas fatty acids represent a hydrophobic tail (Figure 1), which determines phospholipids’ amphiphilic properties and their ability to form a double lipid layer within cell membranes and organelles (Figure 2). Phospholipid-based bilayers in complexes with various membrane-bound proteins allow a cell to regulate membrane permeability for signal molecules, metabolites and ions. The phosphate group can be modified with simple molecules such as choline, ethanolamine or serine. The major phospholipids are glycerophosphatides (phosphatidylcholine, phosphatidylethanolamine, phosphatidylserine, phosphatidylinositol, phosphatidylglycerol, phosphatidylglucoside, cardiolipin) and phosphosphingolipids–sphingomyelins (Figure 1). These head groups define physical and chemical properties of an individual phospholipid and membrane structures dependent on the phospholipid content. The relation between physical structure of phospholipids and their function is well described in a recent review [64], and may be the key to lipid metabolism-associated pathologies [65,66,67].

**Figure 3:**
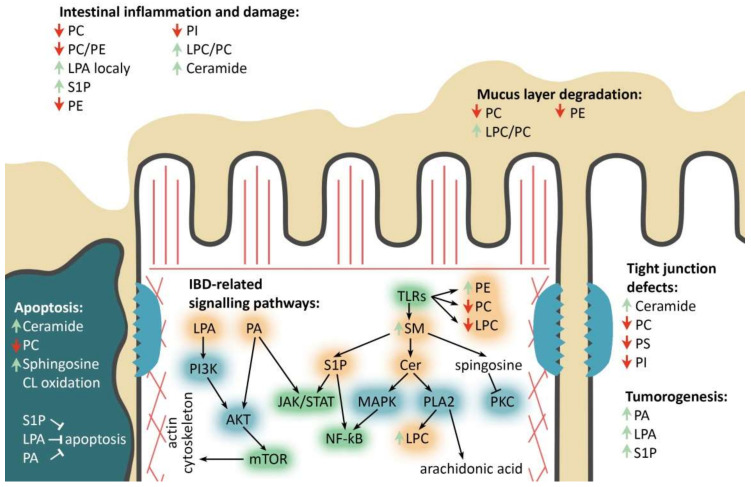
Major phospholipids’ roles in the enterocyte during IBD. PC/PE/PS/PI—phosphatidylcholine/-ethanolamine/-serine/-inositol, LPC-lysophosphatidylcholine, (L)PA—(lyso)phosphatidic acid, SM—sphingomyelin, Cer—ceramide, S1P—sphingosine-1-phosphate, IBD-related signaling pathways: TLRs—toll-like receptors pathway, mTOR—mammalian target of rapamycin pathway, JAK/STAT—Janus kinases/signal transducer and activator of transcription proteins pathway, NF-kB-nuclear factor kappa-light-chain-enhancer of activated B cells. Phospholipids’ remodeling and other involved enzymes: PI3K—phosphoinositide 3-kinase, AKT—protein kinase B, PLA2—phospholipase A2, PKC—protein kinase C, MAPK—mitogen-activated protein kinase. Red and green arrows refer to decrease and increase in corresponding phospholipids, respectively. Black arrows and blind-ended arrows show stimulatory and inhibitory effects in lipids, respectively.

More than 30 IBD-linked loci are involved in JAK/STAT, IL23/Th17, TLR and mTOR signaling pathways [68,69,70,71,72,73]. Specific families of pathogen-associated molecular pattern recognition receptors are responsible for detecting microbial pathogens and generating innate immune responses (Figure 3). Mammalian Toll-like receptors (TLRs) are membrane-bound receptors expressed in innate immune cells, such as macrophages and dendritic cells, and induce responses to the bacterial membrane components, such as lipids, lipoproteins, lipopolysaccharide and proteins [74,75]. Pathogen recognition by TLRs provokes rapid activation of innate immunity by inducing production of proinflammatory cytokines via activation of NF-ƙB and Mitogen-activated protein kinase (MAPK) [75]. TLR-mediated innate immune dysfunction is thought to be one of the central traits in the pathogenesis of IBD [76]. It has been shown that TLR4 activation can significantly affect phospholipid composition ratio in IECs’ membranes [77,78,79].

The inflammatory effects of cytokines involved in the pathology of CD and UC are known to be mediated via Janus kinase (JAK) signaling and Signal transducer and activator of transcription (STAT) families of DNA-binding proteins [80,81]. In mammals, the JAK/STAT pathway appears to be the main signaling mechanism for a wide array of cytokines and growth factors, and JAK inhibitors are efficient in IBD therapies [82,83]. Several studies relate phospholipids to JAK/STAT signaling cascade. For instance, PA has been shown to initiate a rapid phosphorylation of JAK2 and STAT1/3 following production of IL-1β and IL-6 [84]. Sphingosine-1-phosphate (S1P), a signaling sphingolipid metabolite, showed mitochondrial protective effects via the JAK/STAT in cardiomyocyte cell culture [85]. However, a direct relation between phospholipids and this signaling pathway is yet to be discovered in IBD.

Host nucleotide-binding oligomerization domain (NOD)-like receptors include several families of pattern recognition receptors responsible for detecting luminal bacterial components such as flagellin, peptidoglycan and lipopolysaccharide. Importantly, the *NOD2* gene was the first and, to date, the most frequently associated with IBD in humans [86]. Deleterious *NOD2* variants are considered as strong predictors of CD and early onset IBD [87]. Deregulated NOD2 signaling, genetic or functional in nature, has been shown to result in chronic IBD [88]. NOD1 and NOD2, two prototypic NOD-like receptors, activate NF-ƙB and MAPK, cytokine production and apoptosis. Phosphatidylcholine and ceramide were shown to interfere with NF-ƙB signal transduction, which may serve as a possible link between phospholipid metabolism and IBD [89,90].

The mammalian mechanistic target of rapamycin (mTOR) is a highly conserved serine/threonine protein kinase, which exists in two complexes termed mTOR complex 1 (mTORC1) and 2 (mTORC2). mTOR kinase is activated by anabolic signals and plays fundamental roles in regulating lipid biosynthesis and metabolism in response to nutritional triggers [91]. mTORC1 is activated by metabolic status and regulates lipid metabolism along with other basic cellular processes [92,93,94], whereas mTORC2 is sensitive to growth factors and controls cytoskeleton, cell metabolism and survival [95,96]. Activation of the mTOR pathway in IECs has been shown to induce inflammation [97]. Among phospholipids linked to mTORC signaling, phosphatidylinositol (3,4,5)-trisphosphate is involved in inflammation [98]. Similarly, phosphatidic acid acts as an allosteric activator of mTORC1 and activates downstream signaling events [99]. The potential relation between phospholipids and IBD may involve their role in these key signaling pathways that control inflammation and cell death. The importance of phospholipid metabolism in lipid and energy homeostasis has been demonstrated in numerous dietary studies, knockout animal models and in cell culture [66,100,101]. Below we summarize the recent data on the role of known phospholipids in relation to IBD.

### 2.1. Phosphatidylcholine

Phosphatidylcholine (PC) consists of glycerol and fatty acid residues, bearing choline as a head group (Figure 1). PC appears to be the most abundant component of cellular membranes, comprising 40–50% of all cell phospholipids and found mainly in the outer leaflet of the plasma membrane [50]. Intestinal PC derives from the diet or from biosynthesis in the liver and enterocytes, followed by secretion into the intestinal lumen [50,102]. In mammals, the major means of PC biosynthesis are CDP-choline and the (phosphatidylethanolamine N-methyltransferase) PEMT pathways [50,103]. The CDP-choline route, also called the Kennedy pathway, is common for PC biosynthesis in all nucleated mammalian cells [50]. Upon entering the cell, choline is immediately phosphorylated by the cytosolic enzyme choline kinase (CK) to phosphocholine. Then, CTP: phosphocholine cytidylyltransferase converts phosphocholine to CDP-choline. Finally, two integral membrane enzymes of the endoplasmic reticulum (ER), CDP-choline:1,2-DAG cholinephosphotransferase (CPT) and CDP-choline:1,2-DAG choline/ethanolamine phosphotransferase (CEPT), transfer phosphocholine from CDP-choline to diacylglycerol (DAG), thereby generating PC [50]. It is important to note that choline is essential to most mammalian tissues, and must be obtained from the diet [104]. The PEMT pathway is mainly specific to liver. In this pathway, PE is methylated to PC via three sequential methylation reactions by PEMT enzyme [103]. Liver-derived PC is secreted into bile and, together with enterocyte-derived PC, forms the continuous lamellar layer in the apical mucus [105].

Digestion of PC by phospholipase A2 (PLA2) results in the synthesis of lysophosphatidylcholine (LPC) and arachidonic acid, both being important bioactive molecules. Phospholipase B (PLB) deacylates intracellular PC to glycerophosphocholine [106]. Phosphatidylcholine-specific phospholipase C (PC-PLC) catalyzes the hydrolysis of the ester linkage between glycerol and phosphate in PC and other phosphatides, such as sphingomyelin (SM) and phosphatidylethanolamine (PE). Hydrolysis of PC by PC-PLC results in the production of phosphocholine and DAG, a well-characterized lipid secondary messenger molecule [107]. Phospholipases D (PLDs) hydrolyzes the diester bond of PC to generate phosphatidic acid (PA), choline, and free fatty acids [104,108].

PC is also the major bioactive phospholipid found within the gastrointestinal tract [109]. Intestinal epithelial cells secrete mucus, which contains membrane-anchored negatively charged mucin-like glycoproteins and continuous lamellar layer of PC in the apical mucus [110,111]. The hydrophobic lining of the luminal surface plays important functional roles. It prevents microorganisms from contacting the IECs plasma membrane and protects the mucosal epithelium from chemical and mechanical injuries [112,113]. In IBD, there is significant decrease in the amount of mucus and a substantial change in its composition [114]. In particular, as was shown in UC patients, PC content decreases, thereby reducing hydrophobic and protective properties of the mucus [49,115]. Human clinical trials designed to restore colonic PC concentrations in UC have shown promising results [116,117,118]. Downregulation of PC upon colitis may occur due to its increased degradation via PLA2 activity, which is known to increase in patients with UC and CD [119,120]. Consistent with this hypothesis, in a model of experimental colitis, administration of an extracellular PLA2 inhibitor suppressed the development of colitis and positively affected intestinal permeability [121]. Mucosal PLs are also susceptible to phospholipases of bacterial origin. In the stomach, *Helicobacter pylori* colonizes the mucus layer producing phospholipases A1, A2 and C, and can utilize host phospholipids [122,123]. Commensal GI microbial species of *Bacteroides* and *Firmicutes* harbor multiple phospholipases aimed to release PC-derived choline and PA [124,125]. Thus, alteration of microflora upon intestinal inflammation may play an important role in the bioavailability of phospholipids during IBD.

As a result of PC hydrolysis, LPC and fatty acids such as arachidonic acid are formed [126]. Although LPC may be toxic to the intestinal mucosa, the data on the role of arachidonic acid in IBD is more complex [127,128]. Although it was reported to have an ameliorating effect on the intestine [129,130], it was also shown to activate inflammatory pathways, which may contribute to the enhancement of colitis [131,132]. Moreover, upon IBD the composition of the phospholipids in the intestinal mucus changes. In UC, the LPC/PC ratio increases regardless of disease severity [49]. Similarly, the ratio of saturated/unsaturated fatty acids increases favoring arachidonic acid over linoleic and α-linolenic acids during acute stages of CD and UC [133,134]. One of the dietary therapies for IBD is to replace nutritional saturated fatty acid phospholipids with their unsaturated counterparts [135,136].

Alternatively, deregulated biosynthesis of PC may itself be the origin of IBD given its important role as a membranous organelle-forming unit. As mentioned previously, Kennelly and colleagues demonstrated that cytidine triphosphate (CTP): phosphocholine cytidylyltransferase-α knockout results in spontaneous colitis in mice [51]. The ER stress caused by the lack of PC blocks mucin biosynthesis and secretion via vesicular transport and causes Goblet cell necroptosis. The authors further speculate that the colon largely relies on de novo PC synthesis to maintain membrane lipid composition in contrast to the small intestine, where PC is absorbed from the diet and bile. Therefore, colonic epithelium may be more sensitive to lipid homeostasis. Despite a number of theories, the mechanism behind an ameliorating effect of PC on IBD is not fully understood. There is a report suggesting its ability to inhibit inflammatory responses caused by TNF-α, as revealed in cultured cells using NF-ƙB activation and inflammatory gene expression as a read-out [137]. The authors propose that PC may change the properties of cell membranes and interfere with signal transduction, resulting in the reduced inflammatory response. At the same time, PE and SM have not shown any anti-inflammatory effects even though they might also affect cell membranes and can potentially attenuate signal transduction [137]. As phospholipids have distinct physical and bioactive properties depending on their chemical structure, they may differentially affect lipid raft formation and phase separation processes that are key to signal transduction and cell–cell interactions. The role of individual phospholipids in these processes is yet to be investigated.

### 2.2. Phosphatidylethanolamine

Phosphatidylethanolamine (PE) contains an ethanolamine as a nitrogenous head group attached to the phosphate residue (Figure 1). PE is the second most abundant phospholipid in mammalian cells, providing 20–50% of total membrane phospholipids [138]. PE is normally enriched within the inner leaflet of the plasma membrane (Figure 2). In the mitochondria membranes, PE represents about 40% of all phospholipids, and is especially abundant in the inner membrane, making up about 15–25% of total phospholipids in other organelles [139]. In addition to its role in the formation of membranous structures, it is involved in protein biosynthesis [140,141], mitochondrial respiration [142] and autophagy [143]. Moreover, it serves as a substrate for PC and phosphatidylserine (PS) biosynthesis [139,144].

PE is abundant in food, with some variations in formulation depending on the type of product and imported to the cell by mechanisms that are not fully defined to date [50,145]. The two main pathways used by mammalian cells for PE biosynthesis are the CDP-ethanolamine pathway in the ER and the phosphatidylserine decarboxylase (PSD) pathway in mitochondria [146,147,148]. Small amounts of PE can also be synthesized via base-exchange reaction in the ER, catalyzed by phosphatidylserine synthase-2 (PSS2), which replaces the serine residue of PS with ethanolamine [149].

Thus, phospholipid biosynthesis is highly interrelated—PS is formed from PE by phosphatidylserine synthase-1 (PSS1) and PSS2 enzymes [150], whereas PS undergoes decarboxylation to form PE [151]. In turn, PC is synthesized by methylation of PE with S-adenosylmethionine [152,153].

PE is the second most abundant phospholipid within the mucus content and together with other phospholipids, provides mechanical protection of IECs and underlying tissue [137]. Lipidomic profiling of UC patients showed a significant increase in PE in the mucosa [33]. On the contrary, another study found no association between PE and IBD [154]. Administration of PE showed no effect on the inflammatory response induced by TNF-α [89]. In IEC cell lines exposed to pathogenic colitis-associated microorganism enteropathogenic *Escherichia coli*, ethanolamine expression is increased in apoptotic cells and assists bacterial adherence [155]. Thus, microbial induction of apoptosis provides an advantage for bacterial adherence to the intestinal mucosa by increasing levels of the PE receptor. Preincubation with anti-PE antibodies inhibited the adhesion of bacteria to human IECs [156].

PE is a major precursor of endocannabinoids that have multiple functions in the intestinal epithelium and in the organism as a whole [157]. In the intestine, the cannabinoid system regulates motility, barrier function, enteric nervous function and mitochondrial respiration, is generally believed to promote an anti-inflammatory response in model systems [158], and is altered in IBD and colorectal cancer patients [159]. PE also plays a crucial role in signal transduction by G-protein coupled receptors, which may be due to its small polar head favoring a conical shape and the formation of restricted aqueous compartments within the membrane [160,161,162]. This role of PE in IBD is not well characterized. The PC/PE ratio defines the structure of the cell membrane, so a decrease in this ratio negatively affects IECs’ membrane integrity [163]. Similarly, an abnormal PC/PE ratio, specifically the decrease in the PE content, negatively affects energy metabolism in mitochondria and impairs cell survival and growth [50]. However, thus far there is no in vivo evidence on the positive role of PE in IBD, either in mouse models or in clinical data.

### 2.3. Sphingomyelin

Sphingomyelin (SM) is composed of a phosphocholine moiety linked to ceramide via a phosophodiester bond (Figure 1) [164]. SM is found in the plasma membrane and some organelles, such as endocytic vesicles and lysosomes [164]. In intestinal mucosa, SM is relatively minor and constitutes less than 10% of all PLs [165]. SM is found across all classes of lipoproteins and has a prominent role as an amphiphilic surface component [166]. Moreover, sphingolipids are known to modulate transmembrane signal transduction that contributes to cell growth, differentiation and apoptosis, and to the integrity of membrane structures [167,168]. Anticancer activity of SM has also been demonstrated in the context of colorectal cancer [169,170]. SM can be supplied by various food sources, such as eggs, soy beans, meat, fish, and milk [171,172]. Dietary SM is metabolized in the intestinal lumen by sphingomyelinases (SMases) and ceramidases, and absorbed as ceramide, PC, sphingosine and fatty acids [166,173]. Alternatively, SM can be synthesized de novo from serine and fatty acyl-CoA by several enzymes via ceramide intermediate in the ER and Golgi apparatus [164,174].

Signaling downstream of several inflammatory cytokines and growth factors is known to induce SM hydrolysis, resulting in the synthesis of biologically active molecules: ceramide, sphingosine and sphingosine-1-phosphate (S1P) [90,167]. S1P, for instance, is a potent mitogen and inhibitor of apoptosis [175,176,177]. Accumulation of S1P leads to inflammation and tumorigenesis in the colon via NF-ƙB and STAT3 signaling cascades [178,179,180,181,182,183]. On the contrary, sphingosine and ceramide suppress growth and may induce apoptosis [172,177,184,185]. In addition to its contribution to cell cycle regulation, sphingosine is a potent protein kinase C (PKC) inhibitor, which controls a number of signaling pathways associated with inflammation [186,187,188].

Due to its physical properties, long-chain saturated sphingolipids, along with PE and PS, are key constituents of “lipid rafts”—lipid membranous microdomains that serve to anchor proteins involved in signal transduction, membrane trafficking and protein sorting [189]. Proteins modified by saturated fatty acids concentrate within lipid rafts, including G proteins, mitogen-activated protein kinase (MAPK), protein kinase C and others, are often found in lipid rafts [190]. Similarly, lipid rafts provide platforms for TLRs and other receptors to interact with the major inflammatory cytokines TNF-α and IL-1β, or bacterial LPS, and mediate inflammatory response [191].

SM is a component of both cell membranes and mucus in the intestinal tract. SM-enriched dietary supplements have been reported to alleviate the symptoms of IBD in mice due to the inhibition of myeloperoxidase activity [192]. However, opposing data are also reported showing the SM increase in the intestinal mucosa in DSS-induced acute colitis animals [193]. In IBD patients, SM was reported to be decreased in the serum of pediatric CD and in UC, whereas another study reported an increase in SM in the feces of CD patients [32,194,195]. Metabolomic studies in human patients also revealed alteration of ceramide and S1P in CD and UC, respectively [195,196]. It is highly likely that the involvement of SM and its metabolites in inflammatory signal transduction impacts IBD pathology and clinical outcome.

### 2.4. Ceramide

Ceramide is a central molecule in SM metabolism and is crucial for a number of downstream cellular events. Ceramides are composed of a sphingosine and a fatty acid (Figure 1) and serve as important lipid components of the cell membrane and signaling molecules. Ceramides are implicated in cell cycle regulation, biosynthesis and secretion of cytokines, autophagy, inflammation, fatty acid oxidation, etc., [90,197,198,199]. There are three major pathways resulting in ceramide accumulation: de novo synthesis mediated by serine palmitoyltransferase, SM hydrolysis by one of the SMases, and biosynthesis by ceramidesynthase CerS [174,200,201].

In IBD patients, ceramide (d18:1/24:0) and (d18:1/24:2) levels become low in the healed mucosa while being increased as the disease progresses [202]. In a chronic DSS-induced colitis model, ceramide increased by 71%, whereas in the transfer model of colitis it increased up to 160% [203]. Induced by exogenous SMase, ceramide accumulation in tight junctions results in the increase in intestinal permeability [204]. Moreover, addition of increasing amounts of ceramide to the substrate was shown to progressively enhance PLA2 activity [205]. TNF-α has been demonstrated to enhance ceramide production followed by NF-ƙB activation leading to cell apoptosis [206,207], which may slow the healing rate of the mucous damage. Moreover, IBD are not only associated with ceramides, but also with the levels of enzymes controlling ceramide biosynthesis, such as CerS and SMases. SMase stimulates the production of matrix metalloproteinase-1 (MMP-1), which in turn degrades the extracellular matrix (ECM). This results in tissue damage, cell loss and compromised wound healing—all of which are of particular importance in IBD [208]. Increased MMP was reported in ulcerated tissue in IBD patients [209,210].

Ceramides of different chain lengths have distinct health effects. Lack of C14- and C16-ceramides resulted in more pronounced inflammation in a murine model of acute DSS-induced colitis [211,212]. DSS-induced colitis in C16-ceramide deficient mice resulted in enhanced neutrophil infiltration in the colon [212,213]. Increased C14-ceramide was shown to induce ER stress and chronic inflammatory responses, in addition to alter sphingolipid metabolism [214]. The decrease of C24 ceramide levels in genetically manipulated mice reduced the expression of tight junction proteins and induced the phosphorylation of myosin light chain 2, resulting in increased intestinal permeability [211,215].

Intestinal inflammation is known to be accompanied by an increase in ceramide level, and elevated ceramide synthesis is a well-established contributing factor of colitis severity [202,203,216]. Therefore, targeting ceramide biosynthesis may represent one opportunity to alleviate IBD. Indeed, pharmacological inhibition of acid SMase completely abolished the induction of MMP-1 by TNF or IL-1β in Caco-2 cells and human intestinal fibroblasts [203]. Another SMase inhibitor, SMA-7, was shown to suppress ceramide production, NF-ƙB induction, release of inflammatory cytokines by macrophages, and suppress inflammation in DSS-treated mice [217]. Consequently, inhibiting acid SMase may represent a valuable therapeutic strategy for targeting IBD. FTY720, an inhibitor of the S1P receptor, is also of interest [218,219]. This molecule blocks NF-ƙB/IL-6/STAT3 activation and suppresses colitis-associated cancer progression [183]. These strategies may provide new strategies for the treatment of IBD patients, and for colitis-associated colorectal cancer [203,220].

### 2.5. Phosphatidylserine

Phosphatidylserine (PS) is a negatively charged phospholipid molecule in which a hydrogen atom of the phosphate group is replaced by a serine residue (Figure 1). PS content in eukaryotic cells ranges from 2 to 15% [139,221]. In mammalian cells, PS is synthesized in ER membranes and/or mitochondria-associated membranes [222]. In mammals, PS is synthesized by two PS synthases: PS synthase-1 catalyzes the formation of PS from PC, and PS synthase-2 from PE [150]. PS is particularly enriched in the inner leaflet of the plasma membrane [223]. However, during apoptosis, PS translocates from the inner to the outer leaflet of the cell membrane, following inactivation of the translocase and floppase enzymes, in addition to the corresponding activation of scramblase [224,225].

IBD is usually accompanied by an increased apoptosis of IECs [226,227,228]. PS plays a crucial role in this process passing from the inner side of the cell membrane to the outer, acting as a major “eat me” signal of dying cells and nuclei to the phagocytes [229]. Lipidomic profiling of CD patients detected a substantial decrease in total PS and particularly its several types [154]. PS can induce protein kinase C (PKC) [230], and the latter is responsible for a number of cellular responses to various stimuli. Activation of some PKC isoforms, such as iota and zeta, protects against DSS-induced colitis and maintains intestinal barrier integrity [231,232]. Thus, a decrease in the level of PS can affect the activation of PKC and, consequently, the barrier function of the epithelium in the intestine [154]. PS recognition receptors represent a very diverse class grouped according to their ability to recognize the “eat me” phosphatidylserine signal on apoptotic cells, but their functions are not limited to this [233]. Many phosphatidylserine recognition receptors were shown to suppress inflammation by inducing the production of anti-inflammatory mediators during phagocytosis of apoptotic cells [233]. The characteristic property of PS to translocate to the outer membrane can be used for targeted drug delivery to damaged tissues. Thus, Annexin V(A5) (ANXA5) is a calcium-dependent protein that binds to anionic phospholipids (PS, CL, etc.). ANXA5 has been shown to effectively ameliorate TNBS-induced colitis by inhibiting inflammatory cell infiltration [234].

### 2.6. Phosphatidylinositol

Phosphatidylinositol (PI), the basic building block for the intracellular inositol lipids in eukaryotic cells, consists of D-*myo*-inositol-1-phosphate (Ins1P) linked via its phosphate group to DAG (Figure 1) [235]. Within cells, PI is the minor component of the inner membrane leaflet, accounting for less than 10% of all membrane lipids, and its derivatives are even fewer [236]. However, PI derivatives are important secondary messengers, involved in the regulation of various cellular processes including proliferation, cytoskeleton organization, vesicle transport, glucose transport and platelet function [237,238,239,240,241,242]. The glycosyl-phosphatidylinositol serves as an anchor for plasma membrane proteins [243]. PI is synthesized de novo by condensation of *myo*-inositol and CDP-diglyceride via phosphatidylinositol synthase [244,245,246].

The specific molecular structure of PI—five free hydroxyl groups of the inositol ring—allows various signaling kinases (Phosphoinositide 3-kinases, Phosphatidylinositol 4-kinases and Phosphatidylinositol phosphate kinases) to add phosphate groups at positions 3, 4 and 5 of the ring [247]. PI phosphate kinases (PIPKs) do not share significant homology with any other known lipid or protein kinases. Phosphoinositide 3-kinase (PI 3-kinase, PI3K) is known to be the key regulator of the PI3K/AKT/mTOR pathway [248]. The phosphatidylinositol 4-kinases (PI4Ks) synthesize phosphatidylinositol 4-phosphate (PI4P), a key member of the phosphoinositide family. PI4P modulates the membranes of the Golgi and trans-Golgi network (TGN) and regulates trafficking to and from the Golgi apparatus [249]. PI is catabolized via phosphatidylinositol-specific PLC enzymes, which produce two well-characterized secondary messengers: inositol 1,4,5-trisphosphate and DAG. Alternatively, PI is degraded by PLD that hydrolyzes different phospholipids to produce phosphatidic acid and the respective head group residues [237]. Phospholipase-independent mechanisms of phosphoinositide degradation act via dephosphorylation at the D-3, D-4 and/or D-5 positions of the inositol ring by the highly conserved phosphoinositide phosphatases [236,250]. The dephosphorylation of phosphoinositides results in the formation of other signaling molecules that also play important roles [236].

Within the intestinal mucosa, PI along with other phospholipids serve as a surfactant lubricating the intestinal surface and protecting it from physical and chemical irritants [165]. In a rat model of acetic-acid-induced colitis, dietary PI exerted a protective effect on the intestinal mucosa and prevented the development of colitis [251]. Moreover, PI can be used in IBD therapy as a physiological immune suppressor because it can inhibit the inflammatory T-cell response and the secretion of IL-17, IL-2 and IFN-γ, thereby reducing inflammation [252]. Dietary *myo*-inositol was also shown to reduce inflammation and intestinal stem cell activation in both genetic and chemically-induced mouse models of colitis. In a limited clinical trial of colitis patients with a history of recurrent low-grade dysplasia, *myo*-inositol reduced the number of intestinal crypts with activated stem cells [253]. In a model of induced colitis and UC-associated carcinogenesis in mice, inositol has been shown to reduce inflammation and tumor formation by inhibiting signaling via PI3K [254].

Published data further support the involvement of PI3K in IBD. PI3Kδ and PI3Kγ are expressed in leukocytes and play an important role in the innate and adaptive immunity, and therefore are important to understanding inflammatory diseases [255]. PI3Kγ is responsible for the migration of leukocytes from the bloodstream to sites of injury or infection [256]. PI3K regulated the innate immune response in a murine model of UC, thereby controlling colonic inflammation and tumor formation [256,257]. Mice lacking PI3Kγ were significantly protected in the initial state of acute TNBS-induced colitis, however, failed to recover after completion of TNBS administration [258]. Inhibition of PI3Kγ by AS605240 reduced the severity of DSS-induced colitis in mice [259]. Another study also showed that the lack of PI3Kγ prevents leukocyte recruitment and ameliorates colitis in the same model [260]. The critical role of PI3Kγ in inflammatory cell activation and recruitment makes it an attractive target for immunomodulatory therapy [259].

The *PI3Kδ* gene falls to a human IBD susceptibility locus [261]. PI3Kδ-signaling mediates regulatory B-cell differentiation when stimulated with resident microbiota or its components, and is critical for induction and regulatory function of IL-10-producing B-cells [257]. Transgenic mice defective for PI3Kδ subunit p110 develop spontaneous colitis [261]. PI3Kγ-deficient mice had lower incidence of colitis-associated tumors, in addition to reduced tumor number and smaller tumor size [256]. Thus, targeting PI derivatives can be a promising tool in therapy of inflammation and associated carcinogenesis.

### 2.7. Phosphatidic Acid

Phosphatidic acid (PA) is a glycerophospholipid, consisting of a glycerol backbone, two fatty acids and one phosphate group (Figure 1) [262]. It serves as a precursor in the biosynthesis of other phospholipids and fats, but represents less than 1% of total membrane phospholipids [263,264]. Remarkably, PA can play distinct roles within cellular compartments. For example, in the nucleus, PA acts as a mitogen and regulates gene expression, whereas in the Golgi apparatus, it is involved in membrane trafficking [263]. PA can be supplied by many plant foods, being enriched in cruciferous vegetables [265]. Within the organism, PA biosynthesis occurs via three pathways: de novo synthesis from glycerol-3-phosphate, via hydrolysis of PC by PLD and from DAG [262].

PA serves as a substrate for the formation of many other phospholipids via CDP-DAG biosynthesis [264]. In mammals, PA regulates apoptosis and cell proliferation, in particular through the mTOR pathway [265,266]. PA directly interacts with mTOR at the rapamycin-binding domain and can modulate mTOR’s ability to activate downstream effectors [267]. Interestingly, it was shown that only PA containing one or two chains of unsaturated fatty acids can activate mTORC1, which itself is a central regulator of lipid metabolism [91,268]. PA has been shown to reduce aspirin-induced damage to gastric epithelial tissue [265]. Use of the grape exosome-like nanoparticles, which are 98% phospholipids (50% of which is PA), was shown to protect the colonic mucosa from DSS-induced colitis in mice [269]. Similarly, the knockout of PA-degrading enzyme protected mice from inflammation-driven cancerogenesis [270]. However, there is evidence that lisofylline, a functional inhibitor of PA and LPA biosynthesis, significantly reduces inflammation and necrosis in the distal colon in experimental colitis in rats [271]. In addition, deleterious effects of PA are associated with the secretion of proinflammatory cytokines, such as TNF-α, IL-1β and IL-6, and the production of nitric oxide and prostaglandin E2 [272]. Therefore, it is assumed that the above-mentioned properties of PA are associated with lysophospholipase activity that synthesizes LPA from PA [265]. This hypothesis was supported by in vitro and in vivo experiments using gastric PLA2 [265,273].

### 2.8. Lysophosphatidic Acid

Lysophosphatidic acid (LPA) is the simplest phospholipid consisting of a glycerol with a hydroxyl group, a phosphate group and a fatty acid chain. Most LPA fatty acids have long saturated or unsaturated chains [274]. LPA is most abundant in foods such as soy, egg yolk, and cruciferous vegetables [273,275,276]. Biosynthesis of LPA occurs either from PA by phospholipases, or from glycerol 3-phosphate by Glycerol-3-phosphate acyltransferase. Alternatively, LPA can be formed from other lysophospholipids by Autotaxin/lysophospholipase D [274,277].

In the digestive tract, LPA is found in saliva in significant concentrations, and can be formed from PA via gastric and pancreatic PLA2 [265,273,278]. LPA is also released by activated platelets, stimulated fibroblasts, leukocytes, phagocytes and endothelial cells, and mediates a number of cellular responses, such as stimulation of cell proliferation and migration, and regulation of apoptosis [273,279,280,281,282].

LPA acts as a multifunctional lipid messenger in both physiological and pathophysiological processes—wound healing, angiogenesis, tumor growth and the reduction in cholera toxin-induced diarrhea [265,283,284,285,286,287]. In the gastrointestinal tract, LPA facilitates intestinal recovery and wound healing, and prevents stomach ulcers [265,286]. In a course of epithelial damage, LPA stimulates the migration of healthy epithelial cells to the damaged loci and restores the epithelial surface [280]. In cell culture, LPA was shown to induce an immediate calcium mobilization and cytoskeleton remodeling, inducing actin stress fibers and increasing cell migration [279]. Lysophospholipids (lysophosphatidylcholine (LPC), lysophosphatidylethanolamine (LPE), and lysophosphatidylserine (LPS)) are released from platelets at mucosal injury sites by PLA1 or PLA2, followed by conversion to LPA by lysophospholipase D [277]. Therefore, LPA molecule functions as an autacoid that is released locally at the site of injury or inflammation [288].

LPA was shown to prevent intestinal cells from apoptosis via inhibition of the mitochondrial pathway, and from radiation- and chemotherapy-induced apoptosis [281,282]. These protective antiapoptotic effects are mediated by the LPA1 and LPA2 receptors [265,289]. However, an increase in cell proliferation and migration, in addition to inhibition of apoptosis, does not always have a positive effect, because excessive proliferation can lead to tumor formation. Indeed, LPA can stimulate the proliferation of colorectal cancer cells via LPA2 and LPA3 receptors [290]. In IBD patients, expression levels of Autotaxin/lysophospholipase D, an enzyme involved in the synthesis of LPA, gradually increases with inflammation [291]. Administration of the Autotaxin inhibitor, bithionol, reduced experimental colitis, ileocolitis and lymphocyte migration in chemically-induced and in T-cell transfer-mediated colitis mouse models [291]. Thus, further studies of PA and LPA should be focused on the identification of possible mechanisms that contribute to the positive or negative effects of PA and LPA on IBD.

### 2.9. Cardiolipin

Cardiolipin (CL) is the major polyglycerophospholipid in a mammalian cell. CL contains two molecules of PA bridged by a glycerol, comprising four fatty acyl chains and two phosphate groups in total (Figure 1) [292]. CL is localized in mitochondria, mainly on the inner membrane [293]. CL comprises 10–20% of all inner mitochondrial membrane phospholipid content, whereas only about 3% of CL is located on the outer mitochondrial membrane [294,295]. The major functions of CL are closely related to the functions of mitochondria: energy metabolism and electron transport chain maintenance, in addition to stabilization and activation of various membrane complexes and metabolite carriers [296,297,298]. Moreover, CL is involved in apoptosis and mitophagy [299,300].

In contrast to other phospholipids synthesized in the ER and then transported to mitochondria, CL is synthesized at the mitochondria inner membrane [301]. PA serves as a precursor, being actively transported from the ER to mitochondria [302]. Several mitochondrial inner membrane-bound enzymes catalyze CL biosynthesis via a phosphatidylglycerol intermediate or via acylation of other phospholipids. After its initial synthesis, CL can undergo physiologically relevant or pathological remodeling in the mitochondrial matrix or ER, respectively [100]. After remodeling, CL contains more unsaturated acyl chains than CL synthesized de novo [303]. In mammals, CL-specific phospholipases have not been identified; therefore, several phospholipases are thought to catalyze the degradation of CL. PLA2 subclass of phospholipases were shown to utilize CL as a substrate, in addition to being regulated by CL [297]. The action of PLD on CL within mitochondria results in the release of glycerol and phosphatidic acids [304,305].

Abnormalities in the formulation and levels of CL are associated with a number of diseases in various organs and systems, including aging, myocardial ischemia-reperfusion injury, cardiomyopathy in Barth syndrome (BTHS), Senger’s disease, inheritable cardiomyopathies and cancer [306,307,308,309,310]. However, we found no data on direct involvement of CL in the pathogenesis of IBD. Association of IBD-concomitant thromboembolism with elevated anti-cardiolipin antibody levels has been reported, which may be a potential serologic marker of thrombosis risk in IBD patients [311,312,313]. Furthermore, it is known that patients with UC have mitochondrial dysfunction, which may play a role in the pathogenesis of IBD [314]. Mitochondria are the main targets for oxidative stress, although the particular mechanisms of how this impacts IBD is not yet understood [315]. In mammalian cells, reactive oxygen species (ROS) are produced via a variety of cellular oxidative processes, including electron transfer via mitochondrial respiratory chain [316]. Oxidative stress has long been known as a risk factor in IBDs and for colitis-associated colorectal cancer [315,317,318]. Therefore, one of the potential links between CL and IBD may be the impairment of mitochondrial metabolism.

Alternatively, CL may be linked to IBD through the regulation of apoptosis [227,228,319]. Apoptosis can be induced through an internal mitochondrial pathway, involving Cytochrome c translocation to the cytoplasm. Normally Cytochrome c is found on the inner mitochondrial membrane in a CL-associated state. In an oxidized state, Cytochrome c catalyzes peroxidation of CL, which reduces the binding of Cytochrome c to oxidized CL and to the inner mitochondrial membrane [320,321,322]. Apoptosis-associated reorganization of mitochondrial lipids leads to an increase in the permeability of the outer mitochondrial membrane, resulting in Cytochrome c and other apoptosis-associated mitochondrial protein release into the cytoplasm. After entering the cytoplasm, Cytochrome c binds to the Apoptotic protease activating factor-1, which eventually initiates caspase-9 and caspase-3 and triggers apoptosis [323]. Thus, an increase in apoptosis observed upon IBD may be mediated by the oxidation of CL in mitochondria due to increased levels of ROS and oxidative stress in these patients. Given that ultrastructural examination of IBD patients demonstrated abnormal mitochondrial membranes, it is likely that CL is indeed involved in the pathogenesis of IBD.

### 2.10. Minor Phospholipids: Phosphatidylglycerol and Phosphatidylglucosides

The overview of the above-mentioned phospholipids in IBD reveals that these molecules may be of particular importance in the context of GI pathologies. To date, the mechanisms of phospholipids’ action have not been fully investigated and they remain overlooked in terms of their therapeutic potential. Even less is known about minor phospholipids such as phosphatidylglycerol (PG) or phosphatidylglucoside (PGlc) (Figure 1). PG is a minor phospholipid and comprises about 1% of animal membranes. In terms of IBD, it was shown that PG is sensitive to intestinal inflammation and dysbiosis [324]. PG positively correlated with the inflammatory markers and LPS biosynthesis [324]. In the keratinocyte cell line, PG inhibited TLR2 and NF-ƙB, which reduced inflammation in a mouse model not directly related to IBD [325]. In cell culture model of inflammation, PG improved mitochondrial activity and inhibited inflammatory response [326]. PG was not detected in the gastric mucosa of healthy people, whereas in patients with chronic atrophic gastritis, the PG level was increased [327]. However, its role in the intestine remains unclear. PGlc contains glucose as a head group (Figure 1) and is primarily found in the outer leaflet of plasma membrane [328]. In GI tissue from a human autopsy, PGlc was found in epithelial and immune cells [329]. As in many glucosylated phospholipids, PGlc is often enriched within lipid rafts or microdomains and participates in multiple cellular events [330]. It has been shown that GlcCer protects intestinal cells in vitro during inflammation [331]. However, no direct relation to IBD has yet been discovered for this phospholipid.

## 3. Discussion

Given the emerging role of phospholipids in IBD, these molecules should be considered as part of new strategies in IBD therapies. These imply supplementation of phospholipids and fatty acids through the diet and pharmacological manipulation of lipid metabolism and remodeling.

Because the dietary approach is an easy-to-use strategy, it has been popular for a long time; for example, inclusion of the LPA-rich cruciferous plants in the diet of gastritis patients [265]. Oral administration of PC has confirmed its positive effect on IBD in clinical trials [116,117,118]. In mice, a dietary supplement of SM or GlcCer has been shown to alleviate the DSS-induced colitis [192,332].

Diet in general greatly affects IBD. For example, an increase in particular saturated fatty acids as a part of the so-called Western diet provokes IBD [333]. The data on polyunsaturated fatty acids-enriched diets, such as omega-3, in terms of gut health is contradictory, ranging from positive to negative effects [193,334,335,336]. Changes in the ratios of individual phospholipids were also found to accompany the ketogenic diet [337]. The ketogenic diet has been shown to alleviate colitis, possibly by increasing *Akkermansia muciniphila* and its metabolites [338]. In the model of induced colitis in mice, the ketogenic diet maintained intestinal barrier function through the modification of gut microbiota as revealed by fecal microbiota transplantation into germ-free mice [339]. Only fractional data is available on how intestinal microorganisms impact lipids, and particularly, phospholipid metabolism of the host. For instance, *Bacteroides fragilis* produce sphingolipids, which regulate homeostasis of host intestinal natural killer T-cells and protect against oxazolone-induced experimental colitis [339]. *Helicobacter pylori* produces a number of remodeling enzymes, including lipase and phospholipase, that can be involved in the metabolism of host fats [122,123]. There are controversial data about the role of *Akkermansia muciniphila* in the regulation of lipid metabolism [340,341]. Recent studies have revealed that *A. muciniphila* is able to degrade host mucin into various metabolites, such as short chain fatty acids, to regulate host glucose and lipid metabolism [342]. A very interesting study revealed that *Escherichia coli* with a single nucleotide polymorphism in the bacterial *lipocalin* gene is highly enriched in feces from IBD patients. This mutant bacterium caused a decrease in LPE levels, intestinal barrier disruption and inflammation in *C. elegans* and in mice [343]. It has been shown that diet-derived components or their composition, such as polyphenols, can modulate GI microbiota, which, in turn, affects phospholipids’ metabolism [344]. Higher levels of polyphenols in the diet can influence the composition of plasma phospholipids and fatty acids, thus increasing PA and reducing n-6 PUFA [345]. Gut microbiota have been shown to affect the mTOR signaling pathway [346], which, in turn, controls lipid metabolism via several mechanisms [92,93,94].

The second promising strategy for IBD treatment is aimed at the regulation of phospholipid metabolism and remodeling. In IBD, not only does the ratio of phospholipids in the intestinal mucus change, but also the fatty acid content of the phospholipids, namely, saturated/unsaturated fatty acids’ tail composition. PC of the mucus usually contains one saturated (palmitic acid 16:0 or stearic acid 18:0) and one unsaturated (oleic acid 18:1 or linoleic acid 18:2) fatty acid [115]. The high level of arachidonic acid and the low content of linoleic and α-linolenic acids in plasma PC are associated with the acute stages of both CD and UC [133,134]. De novo synthesis of phospholipids is followed by their enzymatic remodeling. This results in generation of a wide variety of phospholipids, differing in the fatty-acyl moieties. The fatty acid composition of phospholipids defines their properties and affects cellular functions [347,348]. Given their key role in generating the required content of different phospholipids, the remodeling enzymes might be considered as possible targets for the development of phospholipid remodeling-based therapeutic approaches [349,350].

## 4. Conclusions

It is becoming clear that personalized approaches may be the only way to cure such a complex and multifactorial disease as IBD [351]. The quantity and ratios of major phospholipids, in addition to balanced fatty acid remodeling, seem to play an important role in the severity of the inflammatory process in IBD and may be crucial for the choice of effective therapeutic strategies. Thus, a deeper understanding of phospholipid homeostasis and, as a consequence, membranous organelle structure and function, is required in order to combat various pathological aspects of IBD. Thus, lipidomic approaches applied to human samples and model systems may still be helpful to further understand the pathological signatures of IBD in terms of phospholipid content. Furthermore, functional studies are needed in order to evaluate the physiological relevance of a particular phospholipid or its modifying enzyme as a possible therapeutic target. Our knowledge of the molecular basis behind the role of phospholipids in IECs’ physiology remains scarce. For instance, it is still unclear how phospholipids affect barrier function, which is dramatically impaired in IBD. The mechanisms underlying the interrelation between tight junctions and their anchoring membrane lipid content are still to be fully understood. Similarly, little is known about how phospholipids regulate other membranous structures in healthy intestinal epithelium and during inflammation. Therefore, further studies are required on the particular phospholipid involvement in the pathogenesis of IBD. In addition, systematic evaluation of the effect of the diet on phospholipid metabolism and its interrelation with microbiota offer new directions for future research.

## Abbreviations

IBD—inflammatory bowel disease; UC—ulcerative colitis; CD—Crohn’s disease; IECs—intestinal epithelial cells; PC—phosphatidylcholine; PE—phosphatidylethanolamine; PS—phosphatidylserine; PI—phosphatidylinositol; PA—phosphatidic acid; LPA—lysophosphatidic acid; LPC—lysophosphatidylcholine; CL—cardiolipin; SM—sphingomyelin; DAG—diacylglycerol.
